# *Fli* ^+^ *etsrp* ^+^ Hemato-Vascular Progenitor Cells Proliferate at the Lateral Plate Mesoderm during Vasculogenesis in Zebrafish

**DOI:** 10.1371/journal.pone.0014732

**Published:** 2011-02-25

**Authors:** Chang Zoon Chun, Indu Remadevi, Marcus-Oliver Schupp, Ganesh Vinayak Samant, Kallal Pramanik, George Albert Wilkinson, Ramani Ramchandran

**Affiliations:** Department of Pediatrics, CRI Developmental Vascular Biology Program, Translational and Biomedical Research Center, Medical College of Wisconsin, Milwaukee, Wisconsin, United States of America; Katholieke Universiteit Leuven, Belgium

## Abstract

**Background:**

Vasculogenesis, the de novo formation of blood vessels from precursor cells is critical for a developing embryo. However, the signals and events that dictate the formation of primary axial vessels remain poorly understood.

**Methodology/Principal Findings:**

In this study, we use ets-related protein-1 (*etsrp*), which is essential for vascular development, to analyze the early stages of vasculogenesis in zebrafish. We found *etsrp* ^+^ cells of the head, trunk and tail follow distinct developmental sequences. Using a combination of genetic, molecular and chemical approaches, we demonstrate that *fli* ^+^ *etsrp* ^+^ hemato-vascular progenitors (FEVPs) are proliferating at the lateral plate mesoderm (LPM). The Shh-VEGF-Notch-Hey2 signaling pathway controls the proliferation process, and experimental modulation of single components of this pathway alters *etsrp* ^+^ cell numbers at the LPM.

**Conclusions/Significance:**

This study for the first time defines factors controlling proliferation, and cell numbers of pre-migratory FEVPs in zebrafish.

## Introduction

In vertebrate embryonic development the blood and endothelial cells emerge side-by-side from a hypothesized common precursor termed, “hemangioblast [1], [2].” In zebrafish and *xenopus*, it has been established that transcription factor fli1 is required for both primitive and definite hemangioblast formation [3], and acts at the top of the transcriptional network dictating blood and endothelial cell development. Recently, in zebrafish, a transcription factor of the ets family, ets1-related protein (*etsrp*) has been identified as a key regulator of vasculogenesis [4]. Further, *etsrp* was shown to be required for the formation of myeloid but not erythroid cells [5]. Both *fli1* and *etsrp* are expressed at zebrafish trunk LPM at 10 som when the putative angioblasts are specified from mesoderm cells, and also at 26 som when the axial vessel is formed at the midline [3], [4]. Sufficient numbers of hemangioblasts or angioblasts are necessary to generate a primary axial vessel. Cell proliferation is one means of attaining the required cell number. However, in zebrafish this mechanism has been considered unlikely to contribute substantially to the precursor pool because of the short temporal window in embryonic vasculogenesis (∼7 hours), and proliferation process especially of differentiated cells takes 16–24 h for one cell cycle. Because fli1 acts at the top of the transcriptional network driving blood and endothelial cell development, and is sufficient to induce expression of key hemangioblast genes such as *etsrp* [3], we hypothesized that *fli* ^+^ *etsrp* ^+^ hemato-vascular progenitors (FEVPs) at the 10 som LPM proliferate prior to migration to the midline. To investigate this hypothesis, we performed detailed *in situ* hybridization (ISH) analysis for *etsrp* in zebrafish embryos of early somite stages, and counted *etsrp* cells between 4 and 8 som (∼2 h window), which showed a two-fold increase in number of cells. Next, we performed pulse chase, chemical treatment and genetic mutant experiments to investigate whether FEVPs proliferation occurred at the LPM. Classic bromodeoxyuridine (BrdU) incorporation analysis, phospho-histone3 immunostaining and hydroxyurea (HU) (S-phase inhibitor) treatment showed that FEVPs are proliferating at the LPM. Further, we observed reduced numbers of FEVPs in *gridlock (grl)* knockdown embryo, which can partly be attributed to reduction in cyclin protein levels. *Grl/hey2*, a Notch target gene was modulated by a variety of approaches, which lead to the expected changes in FEVPs numbers at the LPM. *In vitro*, *grl* knockdown (KD) in differentiated venous endothelial cells (ECs) resulted in minimal changes in cell cycle. Finally, we determined that modulating each component of the Shh-VEGF-Notch-Hey2 signaling axis results in altered numbers of FEVPs indicating that these signals control precursor cell number. Also, we present evidence suggesting that the hypochord, a transient midline structure is the source of VEGF. Together, this study identifies that the canonical Shh-VEGF-Notch-Hey2 signaling axis as responsible for FEVPs behavior, and generation of appropriate numbers through proliferation in a developing vertebrate embryo.

## Results

### Distinct *etsrp* ^+^ cell behavior in head, trunk and tail of embryonic zebrafish

**Figure 1:**
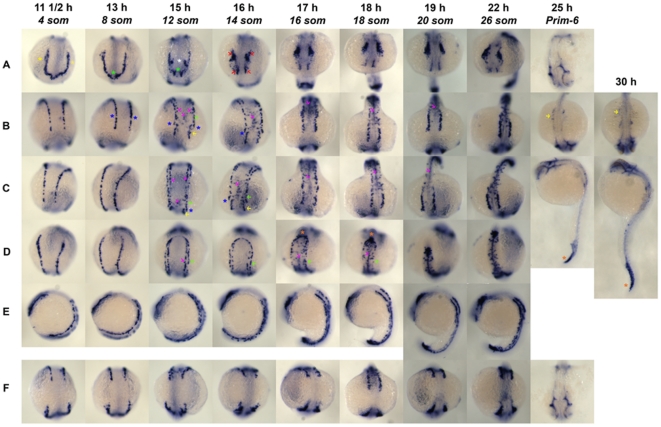
*Etsrp* Whole Mount In Situ Hybridization. A montage of different embryonic stages starting from 4 som to 30 hpf was captured of DIG-labeled *etsrp* antisense ISH whole mount embryos. Embryonic stages are described in somites (som) or hours post fertilization (hpf). A, head; B, dorsal; C, trunk; D, tail; E, lateral; F, between head and dorsal view.

To investigate vasculogenesis *in vivo*, we performed a detailed ISH for *ets1-related protein (etsrp)*, a marker for vascular and some hematopoietic precursors in vertebrates [4]. We performed *etsrp* ISH on embryos fixed at approximately every two hours in development starting at 4 somites (som) until 30 hours post fertilization (hpf) stage (Fig. 1). We noticed three distinct events in the head, trunk and tail regions of the developing embryonic zebrafish vasculature with two groups within the trunk zone.

First, in the head two lateral stripes of *etsrp* ^+^ cells (Fig. 1A, 4 som, yellow asterisk) are connected in the anterior region with a bridge of cells (Fig. 1A, 4 som). At 12 som, two populations of *etsrp* ^+^ cells one from the anterior bridge (Fig. 1A, 8 or 12 som, green asterisk), and another from the middle part of the lateral stripes (Fig. 1A, 12 som, white asterisk), showed an inward trajectory resulting in four cephalic patches of *etsrp* ^+^ cells (Fig. 1A, 14 som, red arrows) at 14 som. The anterior most cells do not undergo substantial changes as the embryo develops until 26 som, but cells in the posterior patches become consolidated into a confined cluster, and presumably contribute to the head vasculature.

Second, in the trunk (Figs. 1B-C, 4 and 8 som), we noticed two lateral stripes of *etsrp* ^+^ cells evenly distributed on either side of the midline. At 8 som, an asymmetric break is noticed (Fig. 1B, 8 som, blue asterisk) in the cells of the LPM resulting in two *etsrp* ^+^ cell populations that show distinct behavior. The anterior *etsrp* ^+^ cells are present at medial position, but the posterior *etsrp* ^+^ cells are in lateral positions (Fig. 1B-C, 12 and 14 som, compare green arrow to yellow arrow). Beginning at 12 som, right at the time when the head populations showed inward trajectory (Fig. 1A, 12 som), an inner population (Figs. 1B-C, 12 and 14 som, pink arrows) of *etsrp* ^+^ cells arose in the trunk region. These cells are closer to the midline than the lateral *etsrp* ^+^ cells in 8 som, and are also observed in the region where axial vessels are forming (Fig. 1B-C, 12 and 14 som, pink arrows). At 18–20 som, the anterior trunk and the midline *etsrp* ^+^ cells come together (Fig. 1B-C, 18 som and 20 som, arrows) at the site of future lateral dorsal aorta (DA). Over the course of next few hours in development, the posterior trunk *etsrp* ^+^ cells eventually disappear (Fig. 1D, 14–18 som, green arrows). Residual *etsrp* ^+^ expression remains at prim-6 (Fig. 1B, prim-6, yellow arrows) and 30 hpf.

In the tail tip region, a third population of *etsrp* ^+^ cells (Fig. 1D, 16 and 18 som, asterisk) gathers, which occurs around 16–18 som stages. It is noteworthy that the trunk *etsrp* ^+^ populations (Fig. 1C, 14 som, pink arrow) observed at the midline in 14 som are improperly assembled at the midline, and at this time point the tail populations are not congregated at the tip as observed in later time points of 16 and 18 som (Fig. 1D, 16 and 18 som, pink and green arrows). At 18 som, when the tail *etsrp* ^+^ populations are aggregated, the *etsrp* ^+^ cells at midline appear to properly assemble in a linear fashion along the anterior-posterior axis, the future site of axial vessels. At prim-6 (Fig. 1, prim-6, yellow arrow) and 30 hpf (Fig. 1, 30 hpf, yellow arrow), we notice remnants of the tail *etsrp* ^+^ population (Fig. 1, prim-6 and 30 hpf, asterisks). Lateral and dorsal views of the entire process are captured in Figs. 1E and 1F respectively. The dorsal view also illustrates that the head and trunk *etsrp* ^+^ population do not fuse throughout development. Starting at 26 som (Fig. 1D, 26 som) and progressing towards prim-6 (Fig. 1D, prim-6), we noticed a gradual loss of *etsrp* ^+^ marker, perhaps associated with differentiation of the *etsrp* ^+^ lineage.

### FEVPs proliferate at the LPM and midline

**Figure 2:**
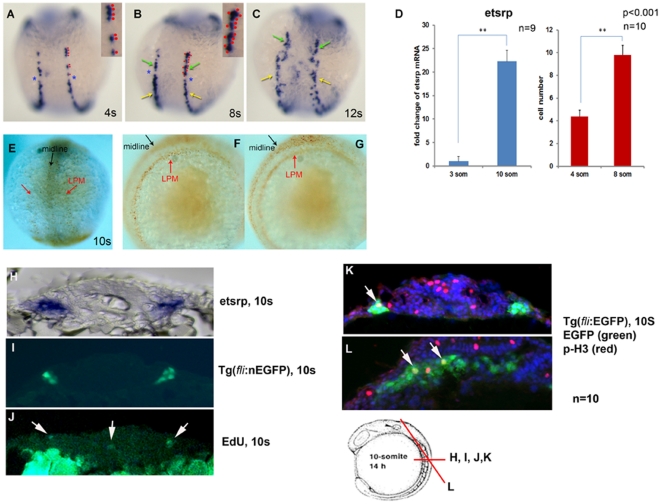
FEVPs proliferate at the LPM. A to C shows dorsal view of *etsrp* ^+^ cells at 4, 8, and 12 som. Two lateral stripes of *etsrp* ^+^ cells evenly distributed on either side of the midline at 4s (A). At 8 som, an asymmetric break is noticed (B, blue asterisks) in the cells of LPM resulting in two *etsrp* ^+^ cell populations that show distinct behavior (B and C, green and yellow arrows). A and B contain insets of *etsrp* ^+^ cells at LPM marked by red dots, which was quantified in panel D, red bars. (D) show quantitation of *etsrp* ^+^ cells as indicated by red dots in panel (B) across each stage indicated in the graph. Error bars represent SEM (qPCR, n = 9; cell count, n = 10). Red bars indicate absolute cell number and blue bars indicate *etsrp* transcript fold change. Samples for analysis were collected at the indicated stages. (E-G) Developing embryos (1 som) were treated with BrdU (10 mM) in embryonic buffer, and subsequent staining was performed with DAB substrate. The BrdU incorporated cells appear in the developing somites at the midline (black arrows) and in the two lateral stripes of *etsrp* ^+^ cells at the LPM (red arrows) in E, F, and G. We confirmed the identity of BrdU^+^ cell at 10 som by ISH with *etsrp* AS probe (H), immunostaining of GFP in *Tg(fli1a:nEGFP)* embryos (I) and EdU incorporation assay (J) post 10 µm cross-section of the embryos at 10 som. White arrows in J show EdU^+^ cells at 10 som. (K) and (L) shows double immunostaining sections of *Tg(fli1a:EGFP)* 10 som embryos stained for EGFP (green), and phospho-histone H3 antibody (p-H3, red). Arrows indicate merged yellow cells stained for both markers. Blue color is from DAPI staining. Cartoon of 10 som embryos with different section plains for panels H-L are indicated.

The observations on embryonic expression of *etsrp* suggest that *etsrp^+^* cells in the head, trunk and tail zones show distinct and coordinated behaviors. We furthermore noted a sizeable increase in *etsrp* ^+^ cell numbers in between 4 and 12 som (Fig. 2A-C). To quantify this observation, we counted the number of *etsrp* ^+^ cells at 4 and 8 som (Figs. 2A & B, inset red dots). These counts showed a 2-fold increase (Fig. 2D, red bar) in absolute numbers of *etsrp* ^+^ cells. We further quantitated whole embryo *etsrp* mRNA level using qPCR. Between 3 to 10 som, we observed a 22-fold increase of *etsrp* ^+^ levels in the embryo (Fig. 2D, blue bar). To examine if the developmental increase in *etsrp* ^+^ pools requires proliferation, we performed bromodeoxyuridine (BrdU) incorporation assay to detect cells undergoing DNA synthesis. Embryos incubated with BrdU from 1 to 10 som showed robust BrdU incorporation at the midline (Fig. 2E), and a weaker signal overlying the presumptive LPM (red arrows, Figs. 2F and 2G). Developing somites serve as an internal BrdU positive control at the midline (black arrow, Fig. 2G).

Since *Fli* is upstream of *etsrp* in the transcriptional hierarchy, and both participate in hematopoiesis and vascular development, we refer to the population of cells in this study as *Fli* ^+^ *etsrp* ^+^ hemato-vascular progenitor cells (abbreviated as FEVPs). These cells are vascular precursor populations, which retain some hematopoietic potential. To identify the proliferating cells in LPM observed in 10 som embryos (Figs. 2F & 2G), we switched from BrdU to fluorescent-conjugated ethynyl deoxyuridine (EdU) because it provides the best option for efficient detection of incorporated EdU. EdU allows for coupling reaction with fluorescent azide dyes, and also allows for use of milder conditions (no DNA denaturation process). We performed IF for green fluorescent protein (GFP) (Fig. 2I) and fluorescent-conjugated EdU (Fig. 2J), incorporation [6] on sections from 10 som transgenic embryos that carries friend leukemia inhibitor (fli-1a) protein promoter driving nuclear localized enhanced GFP (nEGFP) *Tg(fli1a:nEGFP)* [7] in the vasculature. As shown in Fig. 2I, roughly 2–4 GFP^+^ cells are visible in the same location as *etsrp* ^+^ cells in the 10 som ISH embryos (Fig. 2H). Importantly, 1–2 cells out of the GFP^+^ population are EdU^+^ (Fig. 2J, white arrows) suggesting that a sub-population of FEVPs were proliferative. To conclusively determine if the proliferative cell was indeed an endothelial precursor cell, we performed co-staining for phospho-histone H3 (p-H3) and GFP protein in Tg(*fli1a*: EGFP) 10 som section (Fig. 2K). We confirmed these results using double staining for p-H3 and GFP protein on the same Tg(*fli1a*: EGFP) 10 som section (Fig. 2K-L, arrows).

*Etsrp* WISH observations, qPCR message and cell quantitation measurements suggest a rapid increase in this precursor population at the same time that multiple mitotic markers are detected on *Fli* expressing vascular cells. Taken together, these data suggest that FEVPs proliferate at the LPM.

### Expression of the Shh-VEGF-Notch-Hey2 pathway components is consistent with their action at LPM during vasculogenesis

The sonic hedgehog (Shh)-VEGF-Notch-Hey2 signaling pathway is well established for artery vs. vein (A/V) specification during embryonic vascular development in vertebrates [8], [9]. We investigated whether this signaling pathway may be active during vasculogenesis at the LPM. We first investigated whether components of this pathway are expressed appropriately to influence vascular development at the LPM. To determine endogenous *hey2*/*grl* expression, we performed WISH study of *grl* expression during embryonic zebrafish development (Figure S1). *Grl* expression is observed only at the LPM as shown previously [10], and at the midline (Figure S1). Notch1 and Notch4 are the endothelial specific Notch [11]. In zebrafish, *notch1b* expression was intriguingly similar to *etsrp* expression at 10 som in the LPM [12]. ISH for endogenous *shh* (Figure S2A-F) showed that at bud stage (Figure S2C & F) and 3 som (Figure S2B & E), diffuse midline expression was observed, suggesting that presumptive notochord cells express *shh*. At 10 som, *shh* was expressed in midline notochord structures as shown (Figure S2A & D), and demonstrated in previous reports [13]. ISH for *vegf* (both isoforms) showed expression in somites and endoderm at 10 som (Figure S2G, inset & H) [14]. IHC with VEGF antibody clearly showed VEGF protein expression adjacent to hypochord in ventral somites (Figure S2I, yellow arrow). Taken together, all components of the Shh-VEGF-Notch-Hey2/grl pathway are expressed temporally, and spatially at the LPM or in tissues adjoining it during stages in which endothelial precursor cell numbers are generated.

### *Gridlock (hey2)* KD embryos show fewer FEVPs at the LPM

We next focused on addressing whether the Shh-VEGF-Notch-hey2/grl pathway influenced the FEVPs population at the LPM, beginning with the most downstream component, hey2/grl.

**Figure 3:**
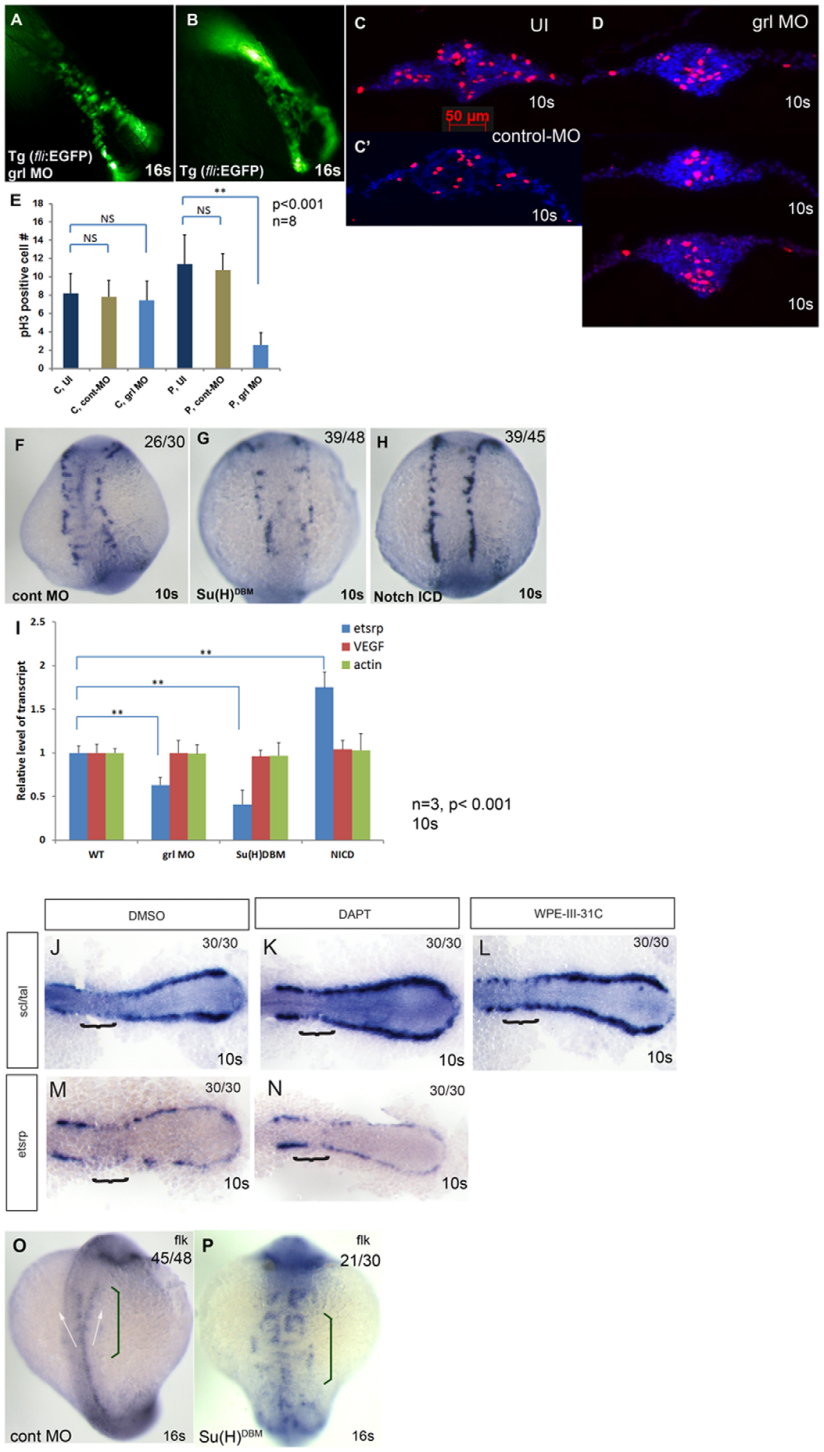
Notch signaling pathway plays a role in FEVPs proliferation at the LPM. Loss of *grl* function by morpholino injection is shown in panels (A) to (D). Panels (A) and (D) are *grl* MO injected, panels (B) and (C) are uninjected fish, and panel C′ is control MO injected fish. In panels (A) and (B), still images of *Tg(fli1a:EGFP) grl*-MO injected (A) or (B) uninjected *Tg(fli1a:EGFP)* 16 som embryos are shown. These images were part of a series of images that were taken under a fluorescence microscope at every 10 min from 15 to 21 hpf, which was reconstituted to movie clips (Movie S1 & S2). The *grl*-MO injected (A) clearly shows fewer *fli* ^+^ cells at 16 som. The same embryo group was also subjected to phospho-histone H3 antibody staining at 10 som (C & D). E shows quantification of number of phospho-histone H3^+^ cell at midline (C: center  =  inside 50 μm bar) in uninjected (UI, dark blue bar), control MO (cont-MO, green bar), gridlock MO (grl MO, light blue bar), and in the same samples at LPM (P: periphery  =  outside 50 μm bar). Error bars represent SEM (n = 8). NS – not significant and ** p<0.001. (F-H) are control MO-injected, *Su(H)DBM* (a gridlock blocker) mRNA-injected and *notchICD* mRNA injected embryos stained for *etsrp* at 10 som. The *Su(H)DBM* mRNA-injected embryo shows dramatic reduction of *etsrp* ^+^ cells at 10 som (F) while *notchICD* mRNA injected embryo (H) shows induction of *etsrp* ^+^ cells at 10 som compared to uninjected embryo (G). (I) qPCR analysis showed that KD of *grl* by MO or dominant negative *(Su(H)DBM)* approach resulted in reduction of transcript level of *etsrp* but not *vegf* and *actin* (Error bar is SEM, n = 3, p<0.001). (J-N) Embryos treated with indicated chemicals from 10–14 hpf, subjected to whole mount ISH for *scl* and *etsrp* markers, and flat mounted. (J-L) ISH for *scl* in posterior LPM of embryos treated with vehicle control DMSO (J) or the γ –secretase inhibitors DAPT (K) or WPE-III-31C [17] (L). *Bracket* indicates the midline convergence segment of the gamma loop of Gering et al. [54] The posterior *scl* expression domain is thickened, and expanded anteriorly in embryos treated with γ–secretase inhibitors. (M-N) ISH for *etsrp* in posterior LPM of embryos treated with vehicle control DMSO (M) or DAPT (N). *Etsrp* expression domain is expanded anteriorly. (O-P) Dorsal view of *Su(H)DBM* mRNA- injected embryo at 16 som (P) showed dramatic mispatterning of *flk* ^+^ cells in trunk compared to that of uninjected embryo (O). (See also Figure S1 and Movie S1 and Movie S2). The numbers on the top right panel of all ISH embryos in this figure indicate number of embryos out of total number displaying that particular phenotype.

Using morpholinos (MOs) and a dominant negative approach, we investigated whether FEVPs cell numbers were affected by loss of grl/*hey2* function. We first injected *grl* MOs into *Tg(fli1a:EGFP)* embryos [15] and observed *fli* ^+^ cells at 16 som (Fig. 3A), and found significantly fewer EGFP^+^ cells compared to uninjected embryos (Fig. 3B). Time-lapse photography of FEVPs migration revealed fewer EGFP cells in the *grl* MO-injected embryo (Movie S2) comparison to uninjected embryos (Movie S1). We also performed p-H3 IF in 10 som control MO (Fig. 3C′), *grl* MO (Fig. 3D), and uninjected embryos (Fig. 3C). We observed no difference in p-H3^+^ cell numbers at the midline, but a decrease in p-H3^+^ cell number is noted at LPM suggesting that *grl* loss of function selectively affects proliferation at the LPM (Fig. 3E).

We next interfered with *grl* expression using a dominant negative Suppressor of Hairless DNA binding mutant [Su(H)^DBM^] construct, which interferes with endogenous Su(H) activity by sequestering Notch intracellular domain (*notchICD*) without DNA binding. [16]. Su(H)^DBM^-injected embryos (Fig. 3G) showed fewer *etsrp* ^+^ cells compared to control MO-injected embryos (Fig. 3F). Taken together, these experiments argue that grl function affects proliferation and numbers of FEVPs cells at the LPM.

### Notch signaling pathway is critical for FEVPs cell development at the LPM

We next investigated Notch, an upstream positive regulator of Grl. Since loss of *grl* function resulted in fewer *etsrp* ^+^ cells, we hypothesized that constitutive activation of Notch dependent transcription by injecting *notch-intracellular domain (notchICD)* mRNA should increase numbers of these cells. Indeed, *notchICD* mRNA-injected (Fig. 3H) embryos showed more *etsrp* ^+^ cells compared to control MO-injected embryos (Fig. 3F). Quantitative determination of *etsrp* transcript levels in *notchICD* mRNA-injected embryo showed a 1.75 fold increase in *etsrp* transcript levels (Fig. 3I, NICD panel). As expected, *grl*-MO and Su(H)^DBM^-injected embryos showed a 0.3 to 0.6 fold reduction compared to WT embryos (Fig. 3I, blue bars). We also checked for changes in *vascular endothelial growth factor* (*vegf*) (Fig. 3I, red bars), and a reference transcript *actin* (Fig. 3I, green bars), and observed no changes in the levels of these transcripts in treated embryos compared to controls. Finally, we inhibited notch ICD generation by chemical inhibition of γ-secretases using DAPT. Compared to DMSO treated embryos (Figs. 3J & 3M), DAPT-treated embryos from 10–14 hpf (Figs. 3K & 3N) showed an overall expansion of *scl* ^+^ cells, and decrease of *etsrp* ^+^ cells most prominently in the posterior LPM resulting in a decreased gap between anterior and posterior LPM (bracket region). Similar results were observed for *scl* ^+^ cells in embryos treated with a second γ-secretase inhibitor, WPE-III-31C- [17] (Fig. 3L) suggesting that *etsrp* ^+^ *scl* ^+^ double positive cells in this region are sensitive to notch inhibition, which is consistent with a recent study that shows co-expression of both markers in this cell population [18]. The whole embryo images of γ-secretase inhibitor-treated embryos are provided in Figure S4A-C. To determine the consequence of notch inhibition, we performed ISH for *flk,* an established marker for differentiated endothelial cells on Su(H)^DBM^-injected 16 som embryos and observed that *flk* ^+^ cells were mispatterned at the midline (Fig. 3P) compared to control MO-injected embryos (Fig. 3O) especially at the region where the future LDA merge (Compare Figs. 3O & 3P, bracket) to form the DA. These results suggest that loss of *etsrp* ^+^ cells at LPM at 10 som results in defective patterning of DA.

**Figure 4:**
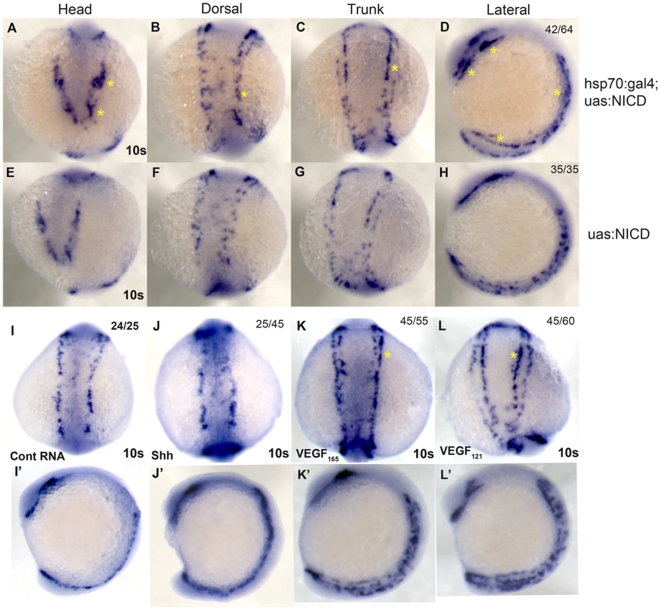
Shh-VEGF-Notch-Hey2 signaling pathway regulates FEVPs proliferation at the LPM. *Etsrp* WISH was performed on *Tg(hsp70:gal4;uas:NICD)* and *Tg(uas:NICD)* embryos. A-H shows *etsrp* ^+^ cells of *Tg(hsp70:gal4;uas:NICD)* and *Tg(uas:NICD)* embryos in head, dorsal, trunk, and lateral. Temporal expression of NICD at 1 som resulted in induction of *etsrp* ^+^ cells at 10 som. I to L show dorsal view of *etsrp* ^+^ cells in uninjected (I), *shh* (J), *vegf_165_* (K) and *vegf_121_* (L) mRNA-injected embryos in trunk at 10 som. I′-L′ show lateral view of the 10 som *etsrp* WISH embryos. *Vegf_165_* mRNA (K) injected embryo shows more *etsrp* ^+^ cells in outer line (yellow asterisk) and *vegf_121_* mRNA (L) injected embryos in inner line (asterisk). (See Figure S2 and S3). The numbers on the top right panel of all ISH embryos in this figure indicate number of embryos out of total number displaying that particular phenotype.

To dissect the temporal requirement of Notch signaling pathway in controlling *etsrp* cell numbers, we utilized the Notch gain-of-function (GOF) transgenic embryo system in which a heat shock-inducible *hsp70:Gal4* transgene drives a Gal4-responsive upstream activating sequence (UAS) *UAS:notch1aICD* allele [19]. Embryos were transferred to 40 °C for 30 min at 1 som, and analyzed using *etsrp* ISH at 10 som. Heat-shock induced embryos showed ectopic induction of *etsrp* ^+^ cells in head (Fig. 4A, yellow asterisk), trunk (Fig. 4B) and tail (Fig. 4C) regions of the developing embryo compared to age-matched *Tg(hsp70:Gal4)* sibling controls (Figs. 4E-G). The induction is pronounced in the lateral view (Figs. 4D & H, yellow asterisks). Collectively, these results argue that the Notch signaling pathway is critically involved in several steps of FEVPs cell development at LPM.

### Shh and VEGF are both involved in increasing *etsrp* cell numbers at LPM

We next investigated the final two upstream elements of the signaling axis, shh and VEGF. We performed *shh* and *VEGF* GOF experiments followed by *etsrp* ISH at 10 som. Shh overexpressing embryos (Fig. 4J) showed an increase in *etsrp* ^+^ cells at LPM compared to age-matched controls (Fig. 4I). We individually injected VEGF_165_, capable of heparin binding [20], [21], and VEGF_121_ mRNA, incapable of heparin binding [22] into 1-cell embryos. Both VEGF_165_ (Fig. 4K) and VEGF_121_ (Fig. 4L) overexpressing embryos showed increased *etsrp* ^+^ cells relative to controls, but distinct cell populations are affected by the different isoforms. To confirm the embryos stages of the injected embryos, an ISH for somite marker *myod* was performed, and is shown in Figure S3A-F (dorsal views), Figure S3G-L (lateral views). VEGF_165_ overexpression (Fig. 4K) resulted in increased numbers in the outer row of cells of trunk LPM, while in case of VEGF_121_ (Fig. 4L, asterisk), the inner row of cells is increased. The lateral views (Figs. 4I′-L′) show more pronounced *etsrp* cell migration defects in VEGF GOF embryos.

**Figure 5:**
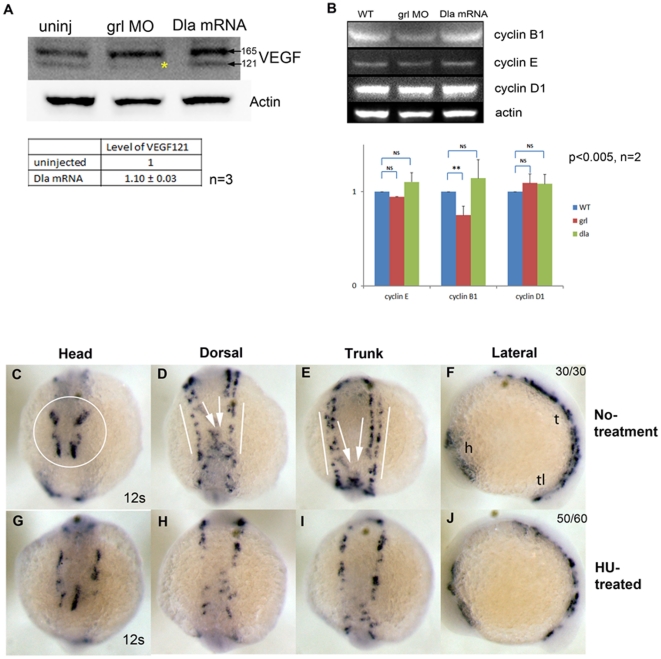
Cell cycle analysis in 10 som embryos. (A) Overexpression of *deltaA* mRNA and KD of *grl* shows complementary changes in protein levels for VEGF_121_ at 10 som. (B) Western blot analysis using total protein from uninjected, *grl* MO, and *deltaA* mRNA-injected embryos showed reduction of cyclin B1 in *grl* MO injected embryos, and slight induction of cyclin B1 in *deltaA* mRNA-injected embryos. No change was observed in cyclin E and D1 levels in *deltaA* mRNA or grl MO-injected embryos. The graph shows quantitation of the western blots with error bars representing SEM (n = 2, p<0.005). (C-J) These panels compare hydroxyurea (HU)–treated (G-J) embryos to untreated embryos (C-F). Treatment of HU, cell cycle inhibitor from 1 to 10 som embryos resulted in reduction of *etsrp* ^+^ cells at the LPM (G-J) at 12 som. In untreated embryos, normal *etsrp* expression patterns were shown as four sets of *etsrp* ^+^ cells in head (C, circle), migrating (D and E, white arrows) and stationary (D and E, white lines) *etsrp* ^+^ cells in dorsal and trunk. h, head; t, trunk; tl, tail in N. (See also Figure S4 and Figure S6). The numbers on the top right panel of all ISH embryos in this figure indicate number of embryos out of total number displaying that particular phenotype.

To explore if temporal and spatial availability of VEGF influenced angioblast numbers at the midline, we injected *deltaA* (*dlA*) (a zebrafish delta homologue/notch ligand) mRNA into 1-cell embryo. *DlA* mRNA has been shown previously to increase floorplate and hypochord cells, sources of VEGF, at the expense of notochord [23]. *DlA* mRNA-injected embryos showed expected increase in *col2a1* expression (data not shown), a marker of floorplate and hypochord cells. In *dlA* mRNA-injected embryos, we noticed a distinct increase (Figure S4G-I) of *etsrp* ^+^ cells compared to control uninjected embryos (Figure S3D-F). We also performed western blots for VEGF protein levels in *dlA* mRNA, *grl* MO and uninjected embryos from 10 som lysates (Fig. 5A). We found that *dlA* mRNA injected embryos showed a slight increase (Fig. 5A, Table) in the faster migrating VEGF_121_ isoform, while *grl* MO showed a decrease in VEGF_121_ isoform (Fig. 5A, lower band missing, asterisk) compared to uninjected embryos. Interestingly, the QPCR data in Fig. 3I showed no change in total *vegf* mRNA (both isoforms) in embryos injected with *grl* MO, while the western blot in Fig. 5A shows changes in VEGF_121_ protein isoform. This suggests that increased stability or presence of *vegf_165_* mRNA isoform compensates for loss of *vegf_121_* mRNA in those embryos. Taking the grl/hey2, Notch, VEGF and Shh data together, it implies that the Shh-VEGF-Notch-Hey2 signaling pathway temporally and spatially regulates *etsrp* ^+^ cell proliferation during embryonic vascular development.

### Grl/hey2 participation in proliferation process in differentiated endothelial cells *in vitro* is minimal

To investigate loss-of-*grl/hey2* function *in vitro*, we designed small interfering (siRNA) using the dicer method [24]. Efficacy of *hey2* siRNA was demonstrated by RT-PCR for *hey2* gene, and showed targeting when compared to control (lacZ) siRNA-transfected cells (Figure S5, hey2 gel). We investigated proliferation of *hey2* silenced ECs initially using human pulmonary artery ECs (HPAECs) because Hey2 functions in artery development [25]. Because HPAECs were difficult to contact inhibit, subsequent cell cycle analysis using FACS was performed on a synchronous population of human umbilical vein endothelial cells (HUVECs). When HUVECs were released from contact inhibition (CI), a robust increase from 4.3 to 21.7% was noticed in S-phase (Figure S5, red bar, CI to CR) along with a concomitant reduction in G0/G1 phase (82.1 to 62.8%, blue bar, CI to CR). In *hey2* siRNA transfected HUVECs (Figure S5, hey2), we noticed an increase in G0/G1 phase (73.3%) compared to *lacZ* siRNA transfected HUVECs (68.6%) and a concomitant decrease in S-phase cells (16.2%-*hey2* siRNA, 17.1%-*lacZ* siRNA). However, statistical analysis showed no significance of this difference across samples. These results argue that *grl* functions in cell cycle events are distinct in endothelial precursor and venous differentiated endothelial cells.

### *Grl/Hey2* affects cell cyclin protein levels *in vivo*, which is reflected in cell phase specific inhibition by chemical inhibitors

To investigate how Grl influences proliferation, we analyzed cell cycle protein levels in zebrafish embryos. We investigated cyclin B1, E and D1 protein levels in *grl* LOF and *dlA* GOF embryos at 10 som (Fig. 5B). *Grl* LOF represents loss of VEGF_121_ and *dlA* mRNA injected embryos represent indirect gain of VEGF function because it is known to increase floorplate and hypochord cells, sources of VEGF. Compared to uninjected WT embryos, *grl* MO embryos showed a decrease in cyclin B1 levels (Fig. 5B, red bar, cyclin B1). In contrast *dlA* mRNA injected embryos showed an increase in cyclin B1 levels (Fig. 5B, green bar, cyclin B1). Little change in cyclin E or cyclin D1 is observed among sample groups.

We next investigated whether chemical inhibition of proliferation would reduce FEVP numbers at stages in which we observed increased *etsrp* expression. Based on published literature for cell cycle inhibitors in zebrafish [26], we treated zebrafish embryos with Hydroxyurea (HU) (S phase inhibitor) [27], Roscovitine and Olomoucine (G1/G2 inhibitor), Genistein (G2 inhibitor) and Colchicine and Nocodazole (M phase inhibitors) at 90% epiboly-1 som and performed ISH for *etsrp* (Figure S6) at 10-12 som. Treatment with HU resulted in fewer *etsrp* ^+^ cells at LPM (Fig. 5H & 5I) and head (Fig. 5G, head, compare white circled area in 5C). In addition, these embryos showed mispatterned *etsrp* cells, suggestive of migration defects (Figs. 5H & I, dorsal & trunk, compare white arrows in 5D & 5E). Roscovitine (Figure S6B), Olomucine (Figure S6C) and Genistein (Figure S6D) treated embryos were indistinguishable from controls. However, Colchicine (Figure S6E) and Nocodazole (Figure S6F) treatment resulted in dramatic changes in *etsrp* ^+^ cell populations in embryos. Taken together, these results indicate that global inhibition of proliferation interferes with the increase in *etsrp* ^+^ cells observed in control staged embryos, suggesting a requirement for proliferation in generation of the appropriately sized FEVP pool.

## Discussion

A recent flurry of publication emphasizes the anatomical and developmental inter-relationship between hematopoietic and vascular cells in vertebrates [28], [29]. Recent studies have shown that that endothelial cells can give rise to hematopoietic precursors [28], [29], and conversely, hematopoietic precursors (HSC) have vascular potential [30]. The shared origin within the LPM for blood and vascular precursors perhaps contributes to this phenomenon [31], [32]. Depending on context, precursor cell populations with vascular and hematopoietic potential can be referred to as hemangioblast or hemogenic endothelium [33]. Because fli1 is the most upstream transcription factor in the hierarchy of factors that drive specification of blood and vascular precursor pools [3], and Scl [34], [35], and more recently etsrp [4], [5] transcription factors (downstream of fli1) operate in both vasculogenic and hematopoietic lineages, we refer in this study to *fli* ^+^ *etsrp* ^+^ hemato-vascular progenitor cells (abbreviated as FEVPs) as vascular precursor populations, retaining some hematopoietic potential. The identification of *etsrp* as a marker required for vasculogenesis in zebrafish [4] and *Xenopus* [36], has allowed the investigation of the early events in vasculogenesis in a developing embryo. Here, we have taken advantage of this marker analysis in developing embryonic vasculature in zebrafish.

In this study, we have demonstrated that FEVPs proliferate at the LPM *in vivo*, and that cellular proliferation is critical for generation of correct numbers of vascular precursors. Several salient features can be surmised from this study. First, *etsrp* ^+^ populations in the head, trunk and tail follow distinct developmental and morphogenetic programs in response to local cues. Second, the Notch signaling pathway regulates FEVPs proliferation at the LPM both temporally and spatially revealing a novel contribution for this pathway in addition to its previously understood role in A/V specification. Third, hypochord/endoderm may be the critical tissue source for VEGF driving FEVPs proliferation at the LPM. Finally, cell cycle protein levels are altered in embryos where Notch signaling is attenuated, suggesting a mechanistic basis for FEVPs proliferation at the LPM.

*Etsrp* expression provides an unprecedented window into the early events of vasculogenesis, as *etsrp* ^+^ populations in distinct regions of the embryo appear to respond to local cues in different manners and generate a pattern that is specific for that region. First, injection of different *vegf* mRNA isoforms *vegf_16_*_5_ vs. *vegf_121_* results in differential effects on *etsrp* ^+^ cells in the trunk and head. Importantly, other groups [37] have reported similar observations at a later time point (30 hpf). Second, HU-treated embryos showed different outcomes in the head and trunk *etsrp* ^+^ cells. Thus the major populations of *etsrp* ^+^ cells along the anterior-posterior axis of the embryo show distinct developmental effects and differential sensitivity to perturbations.

**Figure 6:**
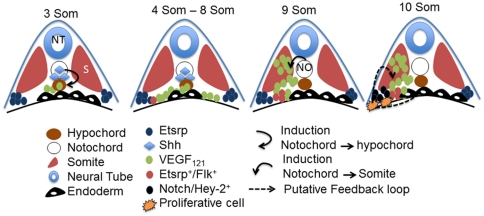
A model depicting the Shh-VEGF-Notch-Hey2 pathway in LPM angioblast proliferation. This model is based on data from here and from other groups [12], [14], [37], [38], [40], [41], [55]. Nomenclature for the various structures, molecules and cell types are indicated. Starting at 3 som, sonic hedgehog (shh) or other signal from the notochord stimulate VEGF_121_ expression from adjoining hypochord, which is associated with endoderm cells lining the yolk boundary. At 4–8 som, VEGF_121_ diffuses via intermediate mesoderm and contacts angioblast cells that are positive for *etsrp* and *flk* marker. At 9 som, a second source of VEGF from somite is detected, which is likely induced by shh from Notochord. Whether the two sources of VEGF have similar or different function is unknown. At approximately 10 som, the VEGF binding to its receptor (Flk?) triggers Notch activation at the LPM [12], which results in activation of downstream target genes such as *hey2/gridlock*. We hypothesize that this signal activation triggers angioblast proliferation at the LPM. A second feedback loop of hey2-VEGF between *hey2* angioblast and VEGF in somite or in hypochord exists, but the temporal and spatial function of this loop is yet to be determined. NO: notochord, NT: neural tube, S: somite.

The Shh-VEGF-Notch-Hey2 signaling pathway has previously been identified as a central player in embryonic vascular development in zebrafish [14], [38], [39]. The expression patterns of Shh-VEGF-Notch-Hey2 are consistent with a role in FEVPs proliferation at the LPM. *Shh* expression is detected as early as bud stage, and continues until 10 som in midline notochord structure as shown by others [13] and us. *Vegf* expression in somite is not seen until 10 som [14], and is observed along the intermediate mesoderm between the LPM and midline at 18–20 som [40]. At 6 som, the *vegf* expression is located in regions adjacent to yolk, and is likely emerging from the transient hypochord structure, which is in close association with the notochord [41]. At 9–10 som, we hypothesize that VEGF from the hypochord (VEGF_121_) diffuses laterally and comes in contact with the LPM cells, which are continuous with the endoderm at this time point. Consistent with this interpretation is our *vegf* ISH and IF data at 10 som. In addition, data in *Xenopus* where diffusible form of VEGF expressed by the hypochord is responsible for angioblast migration [42], and the *dlA* mRNA injected embryo showing more *etsrp* ^+^ cells at the LPM support this hypothesis. Further, notch activation increases hypochord cells at expense of floor plate cells in zebrafish [43], thereby causing an increase in VEGF source at hypochord similar to dlA, and thus explains the putative increase in FEVPs in notchICD-injected and heat-shocked *Tg*(*hsp70:Gal4;uas:nICD)* embryos. The *flk* ^+^ cells are also found at 10 som adjacent to *vegf* ^+^ cells at the LPM [14] suggesting that VEGF-Flk interaction may induce Notch-hey2 signaling cascade resulting in FEVPs proliferation at the LPM. Indeed, of the duplicated Notch1 in zebrafish, *notch1b* expression is intriguingly similar to *etsrp* expression at 10 som in the zebrafish LPM [12] suggesting that Notch1b could transactivate *hey2* promoter [44], and induce a proliferative signal in FEVPs at the LPM. Importantly, our heat shock experiments indicate that precocious activation of Notch at 1 som lead to enhanced *etsrp* ^+^ cell population at 10 som suggesting that pathway components are already in place at the LPM as early as 5 som, assuming that it takes 1–2 h for the production of NotchICD. Finally, Notch target: *hey2/grl* is expressed also at the LPM at 5 som [10] and 10 som indicating that temporally and spatially the Shh-VEGF-Notch-Hey2 signaling axis is likely involved in FEVPs proliferation at the LPM from 3–10 som. A model is presented (Fig. 6) to explain this pathway in the context of FEVPs development from 3 som to 10 som.

Chemical genetic studies with two known γ-secretase notch pathway inhibitors show that *scl* ^+^ *etsrp* ^+^ cell populations are sensitive to notch inhibition thus expanding the junction gap between the anterior and posterior LPMs. These data contradict the recent report from Lee et al, which concluded that Notch signaling functions as a cell-fate switch between endothelial and hematopoietic lineages. This group [45] treated embryos with DAPT at 10–14 hpf, and showed an increase in endothelial cells and decrease in hematopoietic cells at 18–20 hpf. However, this work has several limitations. The interval spanning the drug treatment and final analysis encompass several discrete developmental events at the LPM and midline (Fig. 1). Secondly, the majority of the analysis in this study relies on absolute cell numbers taken from confocal sections. It is clearly not possible to match the exact same location of the section each time across different embryos. Thirdly, taking the first two limitations together, observations comparing absolute numbers from section to section for a process that is undergoing dynamic changes in a 10 h window are necessarily correlative. Our study used two distinct γ-secretase inhibitor treatments, and a host of distinct complementary manipulations to conclude that Notch signaling is involved in proliferation of vascular precursor cells. Combining present work with those presented in Lee et al's, we hypothesize that modulating Notch by DAPT alters the absolute number of precursors at 12 som, thereby changing the numbers of differentiated cells of the blood and vascular lineage observed at 18 hpf. However, this scenario does not account for the selective up regulation of one lineage over another, which is unclear for now.

Although cell proliferation is one mechanism of increasing cell numbers, a second mechanism of cell specification can also achieve the same effect. In this alternate hypothesis, “newly specified endothelial precursor cell in the LPM is the cause for increased cell numbers of the vascular lineage.” At present, we have no evidence to refute this hypothesis. However, the hypothesis in itself does not refute the evidence that is provided here, which shows that endothelial precursor (*fli* ^+^, *etsrp* ^+^) cells are proliferating at the LPM. Therefore, although increased specification vs. proliferation could be reasoned for increasing cell numbers, there is clear evidence here that suggests that once a cell is specified, i.e., *fli* ^+^etsrp^+^ (FEVP), this cell undergoes proliferation. Importantly, the Notch signaling pathway, which we imply here in proliferation shows precedence for playing a functional role in proliferation of midline cells in zebrafish [43], and in zebrafish hematopoiesis [46]. Moreover, Notch also participates in cell proliferation events in Drosophila eye [47]–[48] and wing development [49]. The zebrafish notch study [43] in particular is highly relevant because the authors convincingly show that elevation of notch signaling caused expansion of hypochord at expense of notochord without affecting floor plate cells, and notch inhibition via DAPT caused loss of hypochord cells. They also demonstrated that notch signaling is involved in the proliferation of midline floor plate progenitors similar to FEVPs reported here.

Prior to the mid-blastula transition (MBT), cells of zebrafish embryos cycle without G1 and G2 phases [50], [51]. Duffy and colleagues have reported that key cell cycle regulators involved in S to M phase transition include *ccnb1* (*cyclin B1*), *ccnb2* (*cyclin B2*), and *ccne* (*cyclin E*). Here, we identify that cyclin B1 levels are lower in *grl* LOF 10 som embryos. Enhancement of VEGF production (indirectly by DeltaA) gave the opposite result, which suggests that VEGF-Hey2 signaling axis at 10 som is capable of controlling FEVPs proliferation via modulating cyclin proteins.

This study demonstrates FEVP cell proliferation at the LPM in a developing vertebrate embryo, and implicates the Shh-VEGF-Notch-Hey2 signaling pathway in this process. Whether this process, and the signaling pathway utilized to accomplish precursor cell proliferation are conserved in mammalian development is not known. The data presented here continues the theme of signaling pathways redundantly utilized at multiple stages in a developing embryo, as is often the case in embryonic development [52].

## Materials and Methods

### Zebrafish husbandry and Microinjection

All zebrafish studies were performed according to MCW animal protocol guidelines under protocol # 312-06-2, which were approved by the MCW Institutional Animal Care and Use Committee (IACUC). WT TuAB, *Tg(fli1a:EGFP)* and *Tg(fli1a:nEGFP)* were obtained from ZFIN. All DNA templates were linearized with appropriate enzymes and transcribed by mMessenger mMachine kit (Ambion). Microinjection was performed according to previously established protocols [53]. Seventy-five picograms of each capped mRNA were injected into 1-cell stage embryos. Heat-shock experiment was performed on a clutch of embryos from mating of adult *Tg*(*uas:notch1a-intra*) and *Tg*(*hsp70:gal4*) by transferring embryos at 1 and 5 som to 37 °C for 30 min in a water bath [46]. Subsequently, embryos were cultured at 28.5 °C until 10 som, fixed with 4% PFA, followed by *etsrp* ISH.

### Whole mount *in situ* hybridization, immunohistochemistry, and section

Whole mount *in situ* hybridization (WISH) was performed as described previously [53]. Hybridized embryos were photographed using a Leica Stereomicroscope equipped with a Qimaging camera and images assembled in Adobe Photoshop CS3 Extended. For immunohistochemistry, *Tg(fli1a:nEGFP)* embryo was fixed at 10 som and frozen in TFM. Microm HM 550 (Thermo Scientific, Inc.) was used to generate serial 10 µm cryo-sections embedded in tissue freezing medium (TFM) (Triangle Biomedical Science, Inc.), which was mounted onto glass slide. Immunohistochemistry was performed with rabbit anti-GFP antibody (Cell Signaling, #2555) as primary and goat anti-rabbit IgG-HRP-conjugated as secondary (Cell Signaling, #7074). The signal was detected using tyramide labeled fluorescein (NEN Life Science Products, Inc.) followed by company's protocol. For phospho-histone3 immunostaining, Tg(*fli1a:EGFP*) embryos was fixed at 10 som and processed as described above followed by immunohistochemistry with rabbit anti-GFP antibody (Cell Signaling, #2555) and mouse monoclonal anti-phospho-histone3 (Sigma, H6409) as primary and anti-rabbit IgG-Alexa 488 (Invitrogen, A-21204) and anti-mouse IgG-cy3 (Jackson ImmunoResearch, 715-165-151) conjugated as secondary. The Signals were imaged by Observer Z1 inverted microscope (Carl Zeiss). The details of additional methods used in this study are provided in the supplemental text file (Text S1).

### Statistical analysis

Statistical analysis was performed using the Student's t-test with Graph Pad Prism (GraphPad Software, La Jolla, CA) and Microsoft Office Excel 2010 software package. All data are presented as mean ± SEM (n and p-value are provided in each figure).

## Supporting Information

Text S1 This file contains methods used in the study but not described in the main text file.
(0.06 MB DOC)

Figure S1 A montage of grl whole mount ISH from 8 som to 18 som embryos is shown in panels A-D. A (head), B (trunk) and C (tail) are dorsal views and D (full embryo) is lateral view. The grl expression is observed at 10–12 som at the LPM. This expression is however appears medial and not as lateral as etsrp marker. The expression in the midline starts at 16 som and continues until 18 som. The grl expression resembles flk expression (data not shown) at 17 som and the expression is also seen in axial vessels at this stage.
(1.48 MB TIF)

Figure S2 A-F are whole mount Shh ISH embryos at 10 som (A, D), 3 som (B, E) and bud (C, F) embryonic stage with expression noticed at midline in all stages. (A-C) is lateral view and, (D-F) is dorsal view. (G-H) are whole mount vegf ISH at 10 som. Inset in G shows the vegf expression in endoderm (e) adjoining the yolk and somite (s). I is immunostaining of 10 som section for VEGF protein. Yellow arrow indicates hypochord staining of VEGF protein.
(2.06 MB TIF)

Figure S3 Whole mount ISH with myod probe was performed in 10 som uninjected embryos (A, G) or embryos injected with reagents that are indicated in the panels. A-F is dorsal whole mount ISH view, and G-L is lateral whole mount ISH view.
(1.24 MB TIF)

Figure S4 Whole mount ISH with etsrp AS probe was performed in DMSO (A) and notch inhibitors (B: DAPT or C: WPE-III-31C) embryos at 10 som. Images show the entire embryo at lower magnification. Similarly, etsrp ISH embryos for uninjected and deltaA mRNA-injected embryos are shown in panels D-I. D to F shows etsrp+ angioblasts in uninjected, and G to I shows induction of etsrp+ angioblasts in deltaA mRNA-injected 12 som embryo in head, dorsal, and trunk.
(2.58 MB TIF)

Figure S5 Cell cycle analysis of hey2 siRNA knockdown HUVECs are shown in this figure. (A) RT-PCR for actin and hey2 genes in control siRNA and hey2 siRNA transfected HUVECs are shown. (B) In vitro cell cycle analysis comparisons using synchronized populations of hey2 and lacZ siRNA transfected HUVECs are depicted. KD of hey2 in HUVECS resulted in G0/G1 arrest. The table shows the absolute numbers with +/− SD (n = 3). Comparison across sample groups reflects no statistical significance (NS). CI: contact inhibited, CR: contact released.
(0.67 MB TIF)

Figure S6 Chemical treated embryos at 1 som (10 hpf) were subjected to etsrp ISH at 10 som. Doses and description of inhibitors were based on previous studies in zebrafish [5]. (A) wild type (WT) untreated embryo, (B) Roscovitine, (C) Olomoucine, (D) Genistein, (E) Colchicine, and (F) Nocodazole treated embryo. The cell cycle activity profile for each drug is indicated to the right of each panel.
(3.35 MB TIF)

Movie S1 Utilizing Tg(fli1a:EGFP) embryo and fluorescence microscopy the migration of angioblasts from the LPM to the midline was captured via time lapse imaging every 10 min from 15 hpf to 21 hpf. The images were reconstituted to AVI format movie. Movies clips showing angioblast migration in uninjected (movie 1) embryos were captured.
(1.86 MB AVI)

Movie S2 Utilizing Tg(fli1a:EGFP) embryo and fluorescence microscopy, the migration of angioblasts from the LPM to the midline was captured via time lapse imaging every 10 min from 15 hpf to 21 hpf in grl knockdown embryos. The images were reconstituted to AVI format movie. Movies clips showing angioblast migration in grl-MO injected (movie 2) embryo were captured. KD of grl resulted in much fewer angioblast at the LPM, and mispatterned assembly at midline.
(1.62 MB AVI)
