# Supplementary material for: Fli + etsrp + Hemato-Vascular Progenitor Cells Proliferate at the Lateral Plate Mesoderm during Vasculogenesis in Zebrafish
Source: PLoS One. 2011 Feb 25;6(2):e14732. doi: 10.1371/journal.pone.0014732 (PMC3045372; doi:10.1371/journal.pone.0014732)
Supplement: Text S1 — This file contains methods used in the study but not described in the main text file. (0.06 MB DOC) [file pone.0014732.s001.doc]

**SUPPLEMENTAL TEXT**

**METHODS**

**Reagents**

The *Tg(fli1a:nEGFP)* and *Tg*(*uas:notch1a-intra*), *Tg*(*hsp70:gal4*) were kind gifts from Dr. Nathan Lawson (UMASS) and Dr. Ajay Chitnis (NIH) respectively. *Grl*-MO (PZF1245-9568339) and control MO’s were purchased from Gene-tools, and has been described previously . The cDNA for Dla (Open Biosystems, 6902749), notchICD (gift from Dr. Mayer, Medical College of Wisconsin), Su(H)^DBM^  (gift from Dr. McLaughlin, Tufts University), VEGF_121_ (gift from Dr. Debabrata Mukhopadhyay, Mayo Clinic) and Su(H)^DBM^ (gift from Kelly A. McLaughlin, Tufts University).

**Primer Pairs Used in this study**

Real-time RT-PCR

etsrpF: ACTCTGGGCTGTTCAATCTG

etsrpR: GGTAGGTTTGAGTTGATTCC

vegfF: CTGCTGGTAGACATCATC

vegfR: TTTCGTGTCTCTGTCGGG

zfactinF: GAATCCCAAAGCCAACAG

zfactinR: TACAGAGAGAGCACAGCC

Cloning

ShhF: ATGCGGCTTTTGACGAGAG-TGCTGCTGG

ShhR: TCAGCTTGAGTTTACTGACATCCCCAAAGGATGAAGC

VEGF_165_F: ATGAACTTGGTTGTTTATTTGATACAGTTATTTCTC

VEGF_165_R: TCATCTTGGCTTTTCACATCTGCAAGTTC

**Antisense RNA probe generation and QPCR**

Digoxigenin-labeled antisense RNA probes were synthesized using a DIG RNA labeling Kit (Roche). Antisense RNA probes for *etsrp* (gift from Dr. Sumanas, Cincinnati Children’s Hospital Medical Center), and *flk* were prepared as previously described . The cDNA of VEGF and Shh were cloned into pCR4-TOPO vector post PCR amplification with the respective primer sets (see above). Probes for *flk* and *etsrp* have been described before . QPCR was performed as described previously . The primer pairs for *etsrp, vegf* and zebrafish *actin* are provided above. Total RNA was prepared from 25 embryos at 3 and 10 som using Ambion RNA purification kit and treated with RNase-free DNase. One and half microgram of total RNA was used for 1^st^ strand cDNA synthesis in a final volume of 30 μl according to the manufacture’s protocol (Invitrogen).

**Bromodeoxyuridine (BrdU)/EdU incorporation**

Partially dechorinated embryos were treated with BrdU or EdU in embryonic medium from 1 to 10 som and fixed at 10 som overnight with 4% PFA. For whole mount staining of BrdU incorporated cells, we used BrdU *in-situ* detection kit (BD Biosciences) and followed the company’s protocol with minor modification. EdU treated embryos were cut to 10 µm section and the Click-it EdU Imaging Kits (Invitrogen) was used to detect EdU incorporated cells. EdU and Alexa Fluor dye (C10083) contain the alkyne and azide chemical group respectively, which react together in the presence of copper.

**Chemical Inhibitor Treatment Assay**

All chemical inhibitor treatments of embryos were performed by adding the respective drugs into embryonic buffer containing dechorinated 90% epiboly or 1 som stage embryo and treated embryos were fixed at 10 som. All concentrations used were as reported previously except the concentration for HU, which was 50 mM. For notch inhibitors DAPT (100 μM) and WPE-III-31C (3 μM), treatment was done at 10-14 hpf (3-10 som) and embryos fixed soon after (10 som) for ISH analysis.

**Movie Generation**

At 1 som stage *Tg(fli1a:EGFP)* embryo injected with *grl* MO and uninjected were dechorinated with a pair of fine forceps and mounted in 0.5% agarose (embryo buffer, 45˚C), respectively. The mounted embryo was covered with embryo buffer to prevent drying during time-lapse imaging. The embryo was staged on a fluorescence microscope (Carl Zeiss Inc., Observer. Z1) equipped with incubator module set at 28.5˚C. Under the fluorescence microscope, the time-lapse imaging was performed for 8 hrs with 10 min interval and z-stack of 20 focal planes. The images were reconstituted with software, AxioVision 4.7 and exported to AVI format. Still images from the movie files have been shown in Figs. 3D and 3E.

**Cell Culture, Hey2 siRNA knockdown and Cell Cycle Assay**

Human umbilical vein endothelial cells (HUVEC) were obtained from Lonza and routinely cultured and maintained in endothelial growth medium (EGM, Lonza) under 5% CO_2_ at 37°C in a humidified incubator. All *in vitro* assays were performed using HUVEC from passage numbers 3 to 5. The human *hey2* region corresponding from 31 bp to 481 bp was amplified using primers -forward: GAGAGCGACATGGACGAGAC, reverse: CGCAAGTGCTGAGATGAGAC and the pooled siRNA was generated according to BLOCK-iT^TM^ Complete Dicer RNAi Kit (Invitrogen) recommendations. The effect of *hey*2 gene knockdown on the progression of HUVEC in cell cycle was estimated by flow cytometry. Briefly, HUVEC were contact inhibited in T-75 tissue culture flasks for a period of 6 days during which regular medium was replaced every 2 days. Cells were released from contact inhibition, seeded equally in 100cm^2^ tissue culture dish; and upon attachment were transfected with 250ng of control lacZ and *hey2* siRNA. 12 h post transfection, cells were harvested using trypsin and fixed with 70% ethanol for 45 min. Following fixation, cells were pelleted by centrifugation and stained with propidium iodide (PI 40mg/mL, RNase 100mg/mL) and incubated for 30 min at 37°C prior to FACS analysis.

**Quantitation**

Fig. 2A, B, & D – *etsrp*^+^ cell count at 4 som and 8 som: Post ISH with digoxigenin (DIG)-labeled etsrp probe, *etsrp*^+^ cell was detected with alkaline phosphatase (AP)-conjugated anti- DIG antibody and visualized with AP substrate, BM-purple (Roche). Images of embryos were taken with a stereomicroscope (LEICA MZ16FA), and *etsrp*^+^ cells were counted along the embryonic anterior-posterior LPM axis length at 4 and 8 som stage. The vertical length of the LPM along the embryonic axis for cell count measurement was based on the first *etsrp* stained cell at anterior LPM, and the last *etsrp* stained cell before breakage that is marked with blue asterisk (Fig. 2A and B) at the posterior LPM. Ten of each 4 and 8 som embryos were subjected to *etsrp*^+^ cell count, and error bar represents the standard error of the mean.

Fig. 3C-E – phosphorylated Histone3 (pH3)^+^ cell count: 10 som of uninjected (UI), control MO (n=8) and gridlock morpholino (grl MO) injected embryos were cryo-sectioned in 10 μm (n=8). Post IF with monoclonal anti-pH3 (pSer10) (Sigma, H6409), pH3^+^ cell was counted inside 50 μm at the center (C) and outside 50 μm at the periphery of the body on the section (number of slide=20). For statistical analysis, t-test was applied, and showed significant reduction of pH3^+^ cell in grl MO injected embryos (**p<0.001), while not significant (NS) in uninjected and control MO-injected embryos.

Fig. 3I – Comparison across sample groups were performed with t-test and statistical significance was calculated to **p<0.001, n=3.

Fig. 5A – Quantitation of VEGF121 protein levels were performed by densitometry analysis using ImageJ software. The quantitation is a composite of n=3 and is shown in a table format.

Fig. 5B - Quantitation of the protein band of cyclin B1, cyclin E, and cyclin D1 was performed using a FluorChem ver. 6.0.2 (Alpha Innotech) software, and normalized with actin (n=2). The error bar represents the standard error of the mean, and statistical analysis was determined via t-test with significance calculated to p<0.005.

**Western Blotting**

Cell cycle protein estimation was determined by Western immunoblot analysis. Briefly, zebrafish embryos, 20-30 embryos per experiment group, were lysed using RIPA cell lysis buffer (Sigma). Proteins were electrophoresed using SDS-PAGE (10-12%, Invitrogen) and wet-transferred on PVDF membranes. All blots were blocked with 5% non-fat dry milk in TBST buffer for 1 h at RT and probed with rabbit polyclonal anti-cyclin D1 (Cell Signal), anti-cyclin E (Abcam), anti-cyclin B1 (Abcam), and anti-VEGF (Abcam) antibodies overnight at 4°C. Probed proteins were coupled to HRP-conjugated anti-rabbit or anti-mouse secondary antibodies (Cell Signal) and developed using SuperSignal West-Pico chemiluminescent substrate kit (Pierce Biotechnology).

**SUPPLEMENTAL REFERENCES**

1. Zhong TP, Childs S, Leu JP, Fishman MC (2001) Gridlock signalling pathway fashions the first embryonic artery. Nature 414: 216-220.

2. Zhong TP, Rosenberg M, Mohideen MA, Weinstein B, Fishman MC (2000) gridlock, an HLH gene required for assembly of the aorta in zebrafish. Science 287: 1820-1824.

3. Chun CZ, Kaur S, Samant GV, Wang L, Pramanik K, et al. (2009) Snrk-1 is involved in multiple steps of angioblast development and acts via notch signaling pathway in artery-vein specification in vertebrates. Blood 113: 1192-1199.

4. Sumanas S, Lin S (2006) Ets1-related protein is a key regulator of vasculogenesis in zebrafish. PLoS Biol 4: e10.

5. Murphey RD, Stern HM, Straub CT, Zon LI (2006) A chemical genetic screen for cell cycle inhibitors in zebrafish embryos. Chem Biol Drug Des 68: 213-219.
