# Supplementary figures and images for: Fli + etsrp + Hemato-Vascular Progenitor Cells Proliferate at the Lateral Plate Mesoderm during Vasculogenesis in Zebrafish
Source: PLoS One. 2011 Feb 25;6(2):e14732. doi: 10.1371/journal.pone.0014732 (PMC3045372; doi:10.1371/journal.pone.0014732)

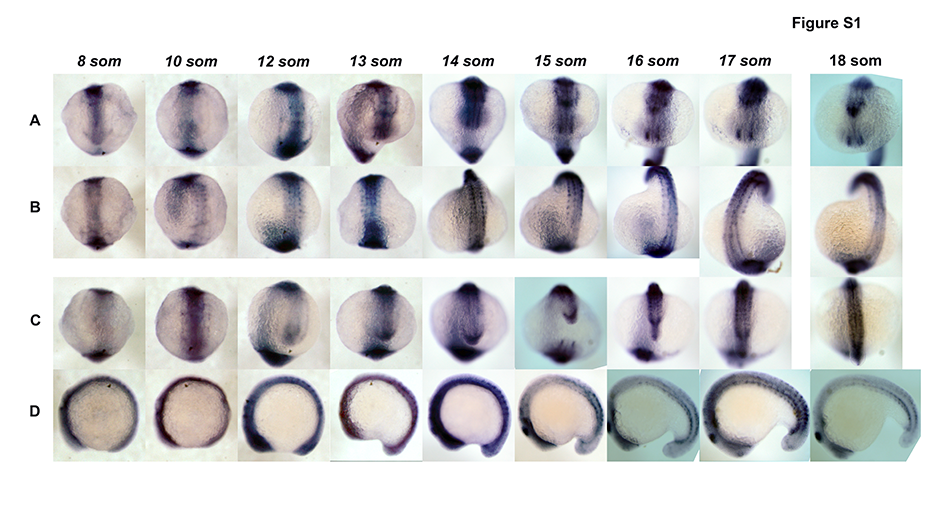

Supplement: Figure S1 — A montage of grl whole mount ISH from 8 som to 18 som embryos is shown in panels A-D. A (head), B (trunk) and C (tail) are dorsal views and D (full embryo) is lateral view. The grl expression is observed at 10–12 som at the LPM. This expression is however appears medial and not as lateral as etsrp marker. The expression in the midline starts at 16 som and continues until 18 som. The grl expression resembles flk expression (data not shown) at 17 som and the expression is also seen in axial vessels at this stage. (1.48 MB TIF) [file pone.0014732.s002.tif]

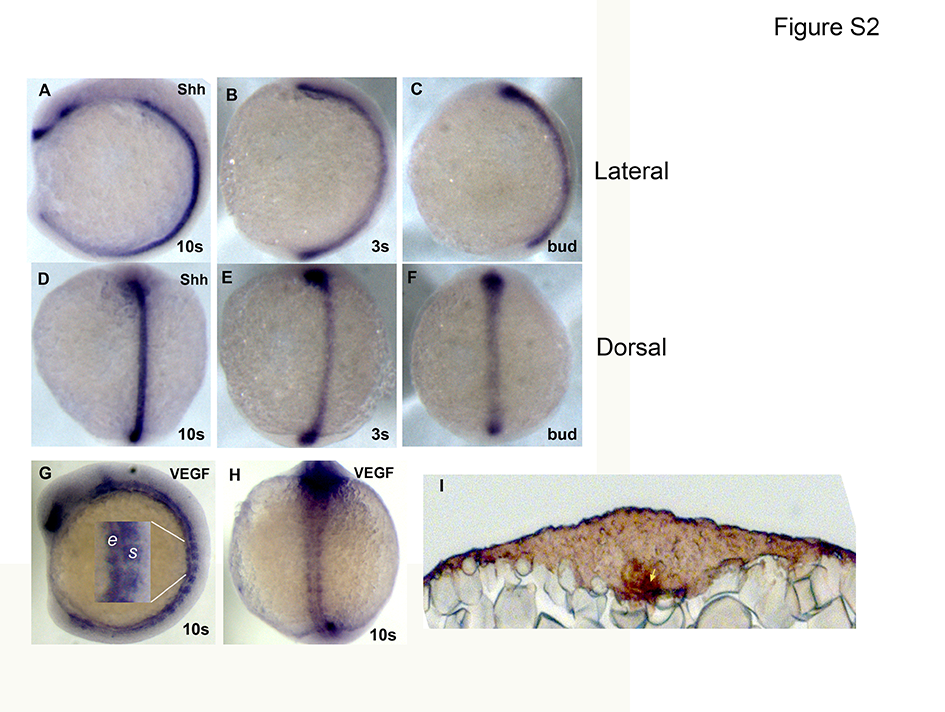

Supplement: Figure S2 — A-F are whole mount Shh ISH embryos at 10 som (A, D), 3 som (B, E) and bud (C, F) embryonic stage with expression noticed at midline in all stages. (A-C) is lateral view and, (D-F) is dorsal view. (G-H) are whole mount vegf ISH at 10 som. Inset in G shows the vegf expression in endoderm (e) adjoining the yolk and somite (s). I is immunostaining of 10 som section for VEGF protein. Yellow arrow indicates hypochord staining of VEGF protein. (2.06 MB TIF) [file pone.0014732.s003.tif]

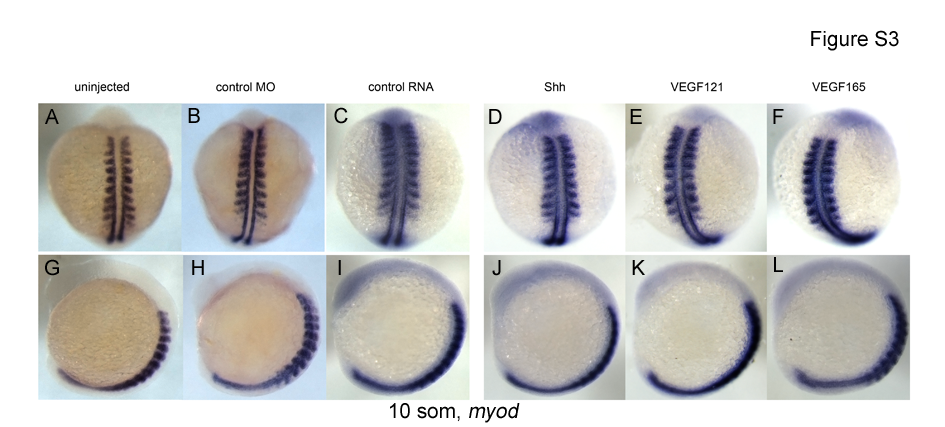

Supplement: Figure S3 — Whole mount ISH with myod probe was performed in 10 som uninjected embryos (A, G) or embryos injected with reagents that are indicated in the panels. A-F is dorsal whole mount ISH view, and G-L is lateral whole mount ISH view. (1.24 MB TIF) [file pone.0014732.s004.tif]

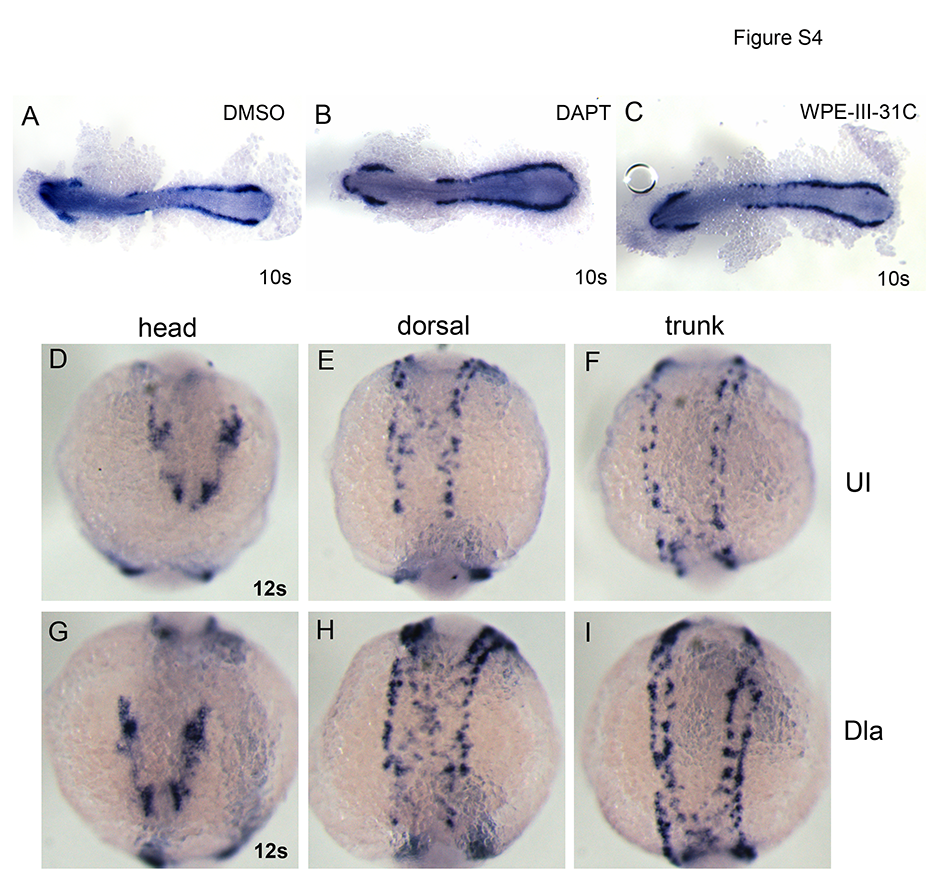

Supplement: Figure S4 — Whole mount ISH with etsrp AS probe was performed in DMSO (A) and notch inhibitors (B: DAPT or C: WPE-III-31C) embryos at 10 som. Images show the entire embryo at lower magnification. Similarly, etsrp ISH embryos for uninjected and deltaA mRNA-injected embryos are shown in panels D-I. D to F shows etsrp+ angioblasts in uninjected, and G to I shows induction of etsrp+ angioblasts in deltaA mRNA-injected 12 som embryo in head, dorsal, and trunk. (2.58 MB TIF) [file pone.0014732.s005.tif]

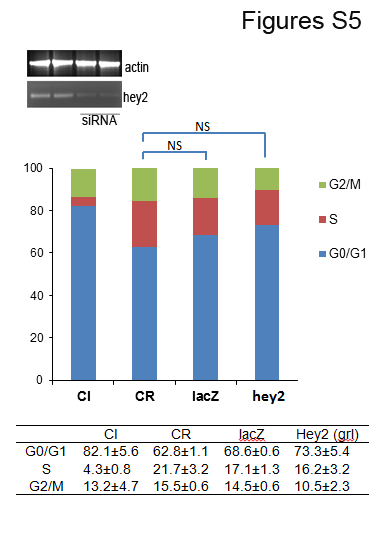

Supplement: Figure S5 — Cell cycle analysis of hey2 siRNA knockdown HUVECs are shown in this figure. (A) RT-PCR for actin and hey2 genes in control siRNA and hey2 siRNA transfected HUVECs are shown. (B) In vitro cell cycle analysis comparisons using synchronized populations of hey2 and lacZ siRNA transfected HUVECs are depicted. KD of hey2 in HUVECS resulted in G0/G1 arrest. The table shows the absolute numbers with +/− SD (n = 3). Comparison across sample groups reflects no statistical significance (NS). CI: contact inhibited, CR: contact released. (0.67 MB TIF) [file pone.0014732.s006.tif]

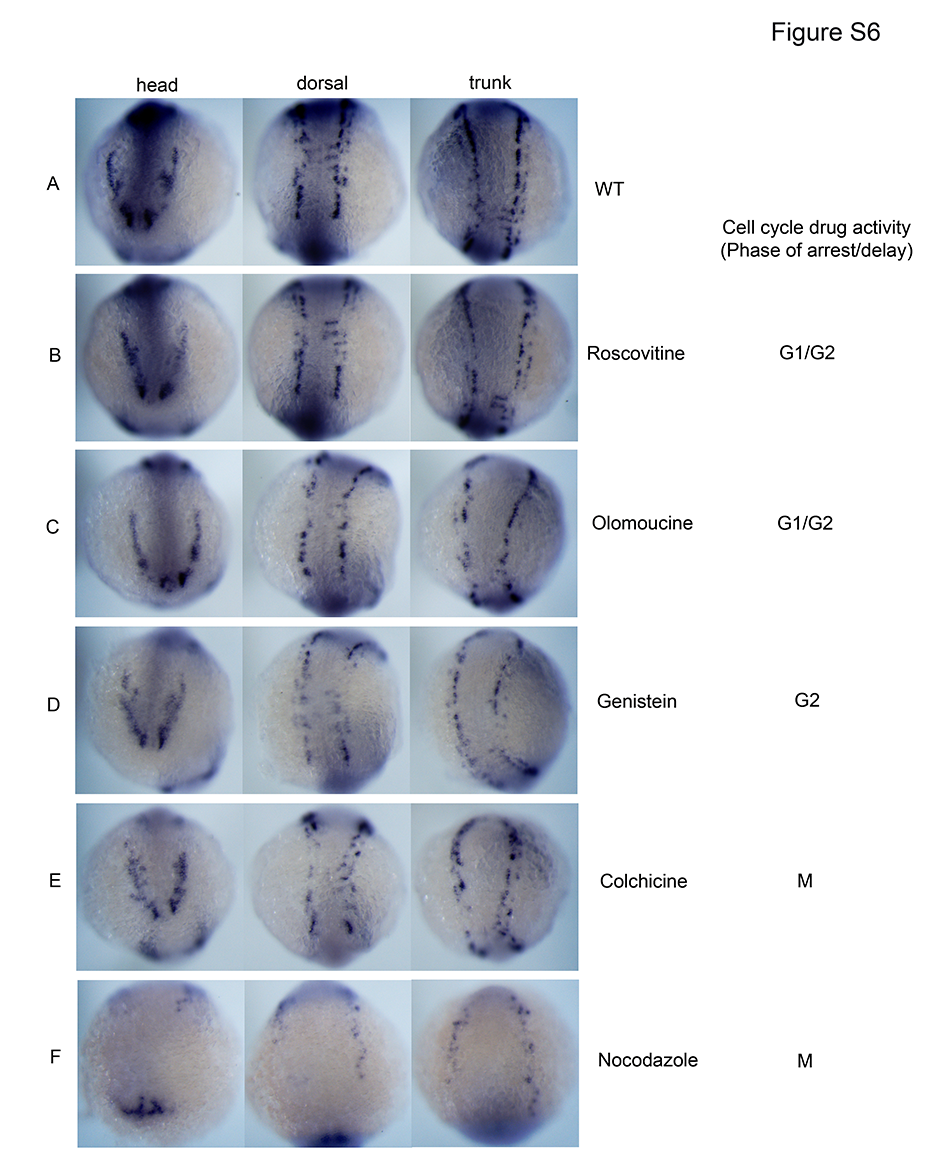

Supplement: Figure S6 — Chemical treated embryos at 1 som (10 hpf) were subjected to etsrp ISH at 10 som. Doses and description of inhibitors were based on previous studies in zebrafish [5]. (A) wild type (WT) untreated embryo, (B) Roscovitine, (C) Olomoucine, (D) Genistein, (E) Colchicine, and (F) Nocodazole treated embryo. The cell cycle activity profile for each drug is indicated to the right of each panel. (3.35 MB TIF) [file pone.0014732.s007.tif]
